# Impacts of gender disparities in mental health and quality of life: A cross-sectional study of Brazilian physicians

**DOI:** 10.1371/journal.pone.0338365

**Published:** 2026-01-12

**Authors:** Marcelo Gobbo Jr, Marina Silveira de Resende, Eduardo Cardoso Moura, Renata de Almeida Pedro, Isabelle Martins Santos

**Affiliations:** 1 Afya Research Center, Afya, São Paulo, São Paulo, Brasil; 2 Afya Educação Médica, Afya, São Paulo, São Paulo, Brasil; 3 Escola Brasileira de Administração Pública e de Empresas – EBAPE/FGV, FGV, São Paulo, Brasil; Jashore University of Science and Technology (JUST), BANGLADESH

## Abstract

**Background:**

Physicians are a professional group at high risk of impaired quality of life (QoL) and mental health due to heavy workloads, exposure to suffering, and structural inequalities. In Brazil, rapid feminisation of the medical workforce has not eliminated gender and regional disparities, and nationwide evidence on physicians’ QoL remains scarce.

**Methods:**

This is a nationwide, cross-sectional, web-based survey of licensed physicians in Brazil between July and August 2024. Eligibility required active registration with a Regional Medical Council and prior use of a digital health platform. QoL was assessed using the WHOQOL-BREF, complemented by items on lifestyle, burnout, depression, and anxiety. Post-stratification weighting aligned the sample with the 2025 Brazilian Medical Census across sex and region. Weighted least squares regression models estimated unique contributions of sex, age, region, career stage, and mental disorders to QoL domains.

**Results:**

A total of 2,005 physicians participated (56.1% women). Weighted WHOQOL-BREF scores post-stratification were 62.2 in the physical, 55.5 in the psychological, 56.5 in the social, and 63.6 in the environment domains, with a global score of QoL (defined as the mean of 0-100 four domains score) of 59.5. Women reported significantly higher prevalence of mental disorders (46.8% vs. 33.5% in men), particularly depression (25.3% vs. 17.7%) and anxiety (39.9% vs. 25.1%). QoL displayed a U-shaped trajectory across the life course, with lower scores in early and mid-career and recovery at older ages. Mental disorders were strongly associated with lower scores across all domains (–9 to –10 points in psychological and QoL, p < 0.001, large effect sizes). Multivariable models identified mental disorder as the dominant predictor of psychological and QoL perception outcomes, while sex explained variance in social relations, and region and age modestly contributed to physical and environment domains.

**Conclusions:**

Brazilian physicians report lower QoL than both the general Brazilian population and international physician cohorts, with particular vulnerability in psychological well-being. Gender disparities persist, with women experiencing greater psychiatric morbidity despite higher social relations scores. Structural inequalities, workload, and dissatisfaction with the health system emerged as key stressors. Interventions should combine structural reforms, gender equity policies, and lifestyle-promoting strategies to support physician well-being.

## Introduction

The World Health Organization (WHO) defines quality of life (QoL) as an individual’s perception of their position in life within the context of cultural values, goals, expectations, and concerns.[1] QoL extends beyond the absence of disease, encompassing physical, psychological, social, and environmental dimensions. To capture these multidimensional aspects, the WHO developed the WHOQOL-BREF, a 26-item instrument validated for cross-cultural use, including in Brazil, where it has shown strong internal consistency (Cronbach’s α > 0.70) and discriminant validity [2,3]

In Brazil, the medical workforce has undergone profound demographic transformations over the past decades. In 2022, the country had 546,171 registered physicians, with women representing 46.6% of the total, a sharp increase compared with previous generations, where the profession was predominantly male.[4] This “feminisation of medicine” is more pronounced among younger cohorts, in which women constitute 61.9% of physicians aged 29 years or younger, compared to 25.2% among those aged 60 or older.[4] Despite these advances, gender inequalities remain evident. Women are disproportionately concentrated in specialties such as Pediatrics (72.8%) and Obstetrics and Gynecology (67.6%), while men dominate surgical and procedure-based fields [4] Income disparities are persistent: even after adjusting for specialty and workload, female physicians earn on average 76% of male physicians’ income.[5]

These structural inequalities intersect with mental health challenges. International evidence shows that physicians face higher prevalence of burnout, depression, and anxiety than the general population. [6,7] In Brazil, studies indicate a growing burden of mental health conditions among medical professionals, especially among women and younger physicians.[4,7] The relationship between QoL and mental health is bidirectional: depression and anxiety impair perceptions of QoL, particularly in psychological and social domains, while lower QoL scores are themselves predictors of poor mental health outcomes [3]. For instance, Cruz et al. (2011) reported that Brazilian individuals with chronic illness and depression scored significantly lower across WHOQOL-BREF domains. Similar findings have been observed among medical students and residents, linking low QoL with high prevalence of depressive symptoms. [8,9]

Moreover, QoL is a dynamic construct that changes throughout the life course. The concept of “response shift” describes how individuals recalibrate their expectations and values when confronted with new health or occupational challenges [10]. For physicians, high workloads, exposure to patient suffering, limited resources, and poor work–life balance often trigger these shifts, affecting both subjective well-being and clinical performance. Female physicians, in particular, report lower QoL due to compounded demands of professional and personal responsibilities, consistent with findings in Thailand and Vietnam [11,12].

Although the WHOQOL-BREF has been extensively validated worldwide [2,13–17], evidence at a national level for Brazilian physicians remains scarce. Previous studies have been restricted to local samples, limiting external validity [18]. A comprehensive national assessment is therefore needed to capture the intersection between QoL, mental health, gender disparities, and occupational determinants in this professional group.

This study aims to evaluate the quality of life and mental health of Brazilian physicians using the WHOQOL-BREF, complemented by lifestyle and occupational measures. Particular emphasis is given to gender disparities and systemic factors, in order to provide evidence relevant for policy making, workforce planning, and physician well-being.

## Methods

### Ethics statement

This study was approved by the Research Ethics Committee of the Instituto de Ensino Superior Presidente Tancredo de Almeida Neves (CAAE 79614324.6.0000.9667; Opinion 2/2024). Data collection was conducted between July 2, 2024, and August 6, 2024. Written electronic informed consent was obtained from all participants prior to inclusion. All procedures complied with the ethical principles of the National Health Council of Brazil (Resolution 466/2012) and the latest version of the Declaration of Helsinki. Reporting follows the STROBE.0-CROSS guidelines.

### Study design and participants

This is a nationwide, cross-sectional, web-based survey of licensed physicians practicing in Brazil. Eligibility criteria included active registration with one of the 27 Regional Medical Councils (CRMs) and prior use or subscription to a national digital health platform. Physicians who declined participation, did not complete the survey, or reported non-medical professions were excluded.

Recruitment was conducted via invitation links distributed through email, in-app notifications, and social media. Data were collected using the QuestionPro™ platform, hosted on ISO 27001-certified servers. A non-random probabilistic sampling method was applied, which may have led to under-representation of physicians in remote or rural areas with limited internet access. To assess sampling bias, the unweighted sample distribution was compared with the 2025 Brazilian Medical Census across sex, macro-region, age band, and medical specialty. The largest deviation was observed in Family Medicine (sample 4.8% vs. census 9.4%), while surgical specialties were slightly over-represented (18.2% vs. 14.5%). Although post-stratification weights were applied to mitigate these imbalances, caution is warranted when extrapolating findings to under-sampled subpopulations.

### Data sources and instruments

#### Quality of life.

Quality of life was assessed using the World Health Organization Quality of Life Questionnaire – Brief Version (WHOQOL-BREF). This 26-item instrument evaluates four domains: physical, psychological, social relationships, and environment. Domain scores were calculated as the mean of their respective items, rescaled to a 0–100 range. The arithmetic mean of the four 0-100 domains scores was calculated and reported as global QoL score.The WHOQOL-BREF has been extensively validated across cultural contexts, with Cronbach’s α ranging from 0.66 to 0.84 across domains in the original WHO multicenter validation [13–15]. In Brazil, Cruz et al. [3] confirmed its reliability with α > 0.70 in most domains and good construct validity in general populations. Studies in Brazilian physicians and healthcare workers also support its internal consistency and sensitivity to occupational stressors [18]. Internationally, validation studies in Iranian healthcare staff [15], Chinese populations [14], and Portuguese-speakers health workers [19] confirm factorial invariance and cross-cultural robustness, allowing meaningful international comparisons.

#### Lifestyle assessment.

A set of five likert-type items was used to assess the frequency of self-reported lifestyle and professional development behaviors. All items shared the same response options varying from never (1) to always (5). The items covered:

- Engaging in regular physical activity (≥150 min/week)
- Meeting one’s own expectations for professional development
- Eating more fresh foods than processed products
- Practicing meditation or mindfulness regularly
- Performing volunteer activities (e.g., NGOs, religious institutions, social foundations, private institutions, or independently

Responses of 4-5 were coded as positive.

#### Burnout, depression, and anxiety.

Mental health indicators were assessed with customized self-report items on burnout, depression, and anxiety. Participants reported presence of symptoms or confirmed diagnoses within the past 12 months or lifetime, as well as their perceived impact on daily life, professional performance, and overall functioning. Instruments such as the DASS-21 were deliberately not included, as our primary outcome was quality of life rather than symptom prevalence. The DASS-21, although psychometrically sound, often reflects a general factor of distress and would introduce conceptual overlap with the psychological domain of the WHOQOL-BREF, complicating interpretation. Moreover, adding 21 items would increase survey burden and risk lower response quality in a web-based nationwide study with physicians, a group already prone to low participation in long questionnaires. From an ethical standpoint, formal symptom screening in anonymous online surveys raises concerns, since positive cases would not have guaranteed referral or follow-up, which goes against recommended practices for mental health screening. Instead, we opted for brief self-report items on burnout, depression, and anxiety, focusing on the perceived functional impact, which better aligns with our study objectives and ensures participant protection.

### Statistical analysis

All analyses were conducted using R version 4.3.3 (survey, car, and stats packages) and SPSS version 29.9. Two-tailed *p*-values <0.05 were considered statistically significant. Weighting procedures. Post-stratification weights were applied using iterative proportional fitting (raking) to align the sample distribution with the 2025 Brazilian Medical Census across sex, macro-region, and age groups. Convergence was reached within six iterations (tolerance = 10 ⁻ ⁵). To reduce variance inflation, weights exceeding 4 were trimmed, following methodological recommendations. This trimming reduced the coefficient of variation of weights from 76% to 49% and decreased the average linearized variance of prevalence estimates by 18%, while minimally affecting point estimates (<0.3 percentage points). All descriptive and inferential statistics presented were calculated with these trimmed weights applied to the full sample [20]. Domain and QoL WHOQOL-BREF scores were calculated and reported in a 0–100 scale.

#### Group comparisons.

Differences by sex and mental disorder status (yes/no) were tested using Welch’s weighted t-tests, with effect sizes expressed as Cohen’s d and Hedges’ g. Differences across macroregions, age groups, and career stages were assessed using weighted least squares (WLS) ANOVA models, with η² and ω² as measures of effect size. Post-hoc pairwise comparisons were conducted with weighted Welch *t*-tests, and *p*-values were adjusted for multiple testing.

#### Multivariable models.

To assess the unique contribution of each predictor to the variance in WHOQOL-BREF domains and the QoL score, WLS regression models were fitted including five predictors: sex, region, age group, career stage, and presence of mental disorder. For each predictor block, the semi-partial R^2^ (ΔR^2^) was computed as the difference between the full-model R^2^ and the reduced-model R^2^ obtained after removing the predictor. Partial R^2^ values were derived as:


$$\frac{R_{full}^{\text{2}}\;-\;R_{reduced}^{\text{2}}}{\text{1}\;-\;R_{reduced}^{\text{2}}}.$$

Omnibus F-tests were used to test the joint significance of all levels of each categorical predictor. Results are presented as ΔR^2^, partial R^2^, F, *p*-values, and percentage of total model R^2^ explained by each predictor.

Exploratory analyses. Non-linear associations between age and quality of life were explored using locally weighted scatterplot smoothing (LOESS), stratified by sex and income quartiles, with 95% confidence intervals.

## Results

### Sample Profile

A total of 2,005 physicians participated. Most participants (56.1%) identified as female, with a heterogeneous distribution by career stage, and a higher prevalence of women among physicians with up to 20 years of practice (Table 1). The Southeast region concentrated nearly half of the sample (49.9%), followed by the South region (20.2%).

**Table 1:** Unweighted sociodemographic and professional characteristics of physicians in the study (n=2005).

Gender	
Female	56.1%
Male	43.6%
Non-binary	0.1%
No answer	0.1%
**Race**	
White	73.4%
Mixed race	21.0%
Oriental	2.6%
Black	2.6%
Brazilian native	0.4%
**Age group (years)**	
< 25	3.8%
26 - 35	51.6%
36 - 45	20.7%
46 - 55	10.6%
56 - 65	7.9%
66 - 75	4.7%
> 76	0.7%
**Marital status**	
Married	52.4%
Single	41.7%
Divorced	5.6%
Widower	0.3%
**Medical degree**	
Graduation	30.5%
Residency	7.7%
Post-graduation	12.7%
Specialist	49.0%
**Time for the academic formation (years)**	
< 5	48.8%
6 - 10	17.7%
11 - 20	12.5%
21 - 30	8.5%
> 31	12.6%
**Average working time range (hours per week)**	
< 44	44.8%
45 - 59	20.0%
60 - 74	24.0%
75 - 89	5.7%
90 - 104	3.7%
> 105	1.8%
**Monthly income (R$)**	
<5k	6.8%
5.001 - 10k	10.5%
10.001 - 15k	21.0%
15.001 - 20k	21.1%
20.001 - 25k	12.9%
25.001 - 30k	9.7%
30.001 - 35k	5.4%
35.001 - 40k	4.2%
40.001 - 45k	2.0%
45.001 - 50k	1.5%
50.001 - 55k	0.7%
55.001 - 60k	0.5%
> 60k	3.5%
**Sample size**	2005

Regarding training and specialization, 30.5% of participants were general practitioners without a specialization, 7.7% were in medical residency, 12.7% were pursuing a postgraduate degree, 20.3% had one specialization completed, 21.4% had two or more specializations completed, and 7.2% were specialists with at least one completed and another in progress.

The average weekly workload of physicians was 50.9 hours, with longer workweeks among men (54.3 hours) and residents (65.7 hours). Most physicians reported working in multiple professionals settings, with a mean of 2.6 different workplaces per professional.

The mean monthly net income was R$ 21,273.00, with a clear gender disparity. Female physicians earned less (R$ 18,343.00) than their male counterparts (R$ 24,960.00). Although this difference was partly explained by a lower workload among women, the disparity persisted when adjusting for hours worked: the mean net income per hour was R$ 113.80 for women and R$ 137.80 for men. This mean difference (R$ 24.00 per hour)was statistically significant (t(1997) = –3.33, p < 0.001). A total of 1,999 physicians were included after weighting by sex, region and age groups using post-stratification (raking) to align the sample with national physician distributions [4].

### Quality of life

Weighted estimates correcting for sex–region imbalance revealed mean scores of 62.19 in the physical domain, 55.46 in the psychological domain, 56.52 in social relationships, and 63.64 in the environment domain, resulting in a global QoL score (mean of the four domain scores) of 59.45 (Table 2). These values represent the best estimates for the Brazilian physician population.

**Table 2:** WHOQOL-BREF weighted domain scores, sex differences, and regional comparisons among physicians (n =1999).

Domain	Mean score	Sex	Cohen’s d	Regional comparison	Eta²
Physical	62.19	p < 0.001	0.24	p < 0.05	0.004
Psychological	55.46	p < 0.001	0.21	p >0.05 (*ns*)	0.005
Social	56.52	p < 0.001	0.3	p >0.05 (*ns*)	<0.001
Environment	63.64	p >0.001	0.08	p<0.05	0.012
Global QoL score*	59.45	p < 0,001	0.14	p >0.05 (*ns*)	0.004

*Mean of the four domains. Cohen’s d interpretation: 0.2 = small, 0.5 = medium, 0.8+ = large effect. Eta² interpretation: 0.01 = small, 0.06 = medium, 0.14 = large effect; ns, not significant.

A consistent upward trajectory of quality-of-life scores with advancing age was observed, with the exception of minor fluctuations in the psychological domain. The youngest group (20–30 years) presented a mean overall score of 52.57, increasing progressively to 78.47 in the 70–80 years group.

The linear regression analysis confirmed a positive association between age and quality-of-life scores. The intercept was 41.19 and the slope 0.35, indicating a mean increase of 0.35 points per year of age (p < 0.001). The model accounted for 3.35% of the variance (R² = 0.0335), with an overall F-statistic of 34.13 (p < 0.001).

### Gender differences

Comparisons by sex using weighted data revealed small but statistically significant differences. Male physicians reported higher scores in the physical (*d* = 0.24, p < 0,001) and psychological domains (Cohen’s d = 0.21, p < 0,001), as well as in the mean of the four domains (*d* = 0.14, p < 0.001). In contrast, women scored significantly higher in social relationships (+6.0 points; p < 0.001, *d* = 0.30, 95% CI 4.22–7.79), while men reported slightly higher scores in environment (–1.5 points; p > 0.001, *d *= –0.10).

### Regional differences

Analysis by macroregion revealed significant variability in the psychological (p < 0.05, η² = 0.005) and environmental domains (p < 0.001, η² = 0.012), although the effects were small. Younger physicians (≤35 years) reported lower Physical and Environment scores compared to those ≥56 years (+3–6 points, *p* < 0.05). In the Physical domain, mean scores ranged from 52.9 in the North to 56.5 in the Southeast (*F*(4,1994)=3.15, *p* = 0.013, η² = 0.006). In the Environment domain, scores ranged from 59.2 in the North to 65.5 in the South (*F*(4,1993)=4.82, *p* < 0.001, η² = 0.010). Differences across regions were not significant for the Psychological, Social relations, or QoL scores.

### Age group

Age-related differences were observed primarily in the Physical and Environment domains. Physicians aged ≤35 years scored significantly lower in the Physical domain than those aged ≥56 years (+3 to +4 points, *p* < 0.05). In the Environment domain, younger physicians (26–35 years) reported lower scores compared to those aged ≥56 years (+4 to +6 points, *p* < 0.05). No significant age-related differences were found in the Psychological, Social relations, or QoL scores.

### Career stage

**Fig 1:**
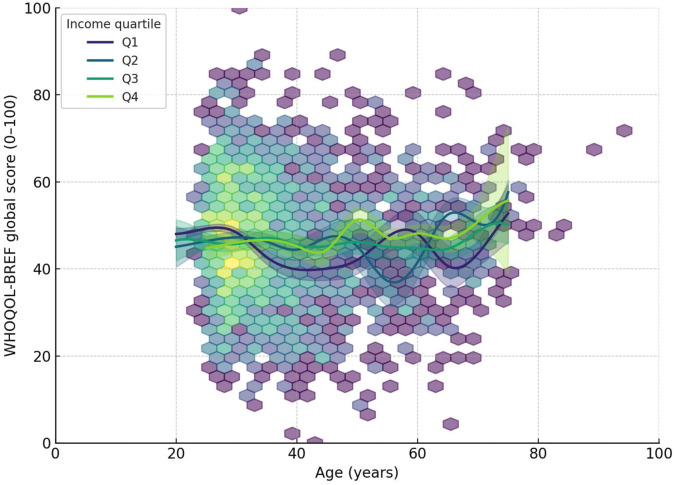
U-shaped age–score association by income quartile. Estimated mean score by age, stratified by income quartiles (Q1–Q4). A U-shaped association is observed across strata. Physicians in Q3–Q4 maintain higher scores, especially in later adulthood, whereas Q1–Q2 remain lower with smaller gains after midlife. Higher values indicate better status.

Physicians with ≤5 years of practice reported lower Physical domain scores than those with >20 years (+3–4 points, *p <* 0.05). No significant differences by career stage were observed in the other domains or in the global QoL score. The graphical analyses confirmed non-linear associations between age and quality-of-life scores (Fig 1).

### Mental disorders

Overall, 39.8% of physicians reported a diagnosis of mental disorder (Table 3), with higher prevalence among women (46.8%) and younger physicians aged 26–35 years (49.6%). Depression was reported by 22.1% of respondents, anxiety disorders by 33.5%, burnout syndrome by 6.7%, and other psychiatric conditions by 6.3%. Depression (25.3% vs. 17.7%) and anxiety disorders (39.9% vs. 25.1%) were significantly more frequent among female physicians compared to males. Burnout and other disorders did not differ significantly by gender.

**Table 3:** Prevalence of self-reported mental health diagnoses among physicians (n = 2005).

Diagnosis of mental disorder	Yes (%)	No (%)
Any mental disorder	39.8	60.2
Depression	22.1	77.9
Anxiety disorder	33.5	66.5
Burnout syndrome	6.7	93.3
Other mental illnesses	6.3	93.7

Self-reported mental disorders (e.g., depression, anxiety, burnout) were strongly associated with lower scores across all domains and in the QoL measure. Physicians with a mental disorder reported substantially lower Psychological (–10 points, p < 0.001, *d *= – 0.70) and QoL scores (–9 points, p < 0.001, *d *= –0.65), as well as lower Physical (–5 points, *d* = – 0.40), Social relations (–4 points, *d* = –0.30), and Environment (–5 points, *d* = – 0.35) scores compared to those without a mental disorder. These represent the largest and most consistent differences identified in the study.

### Impact of stress

**Fig 2:**
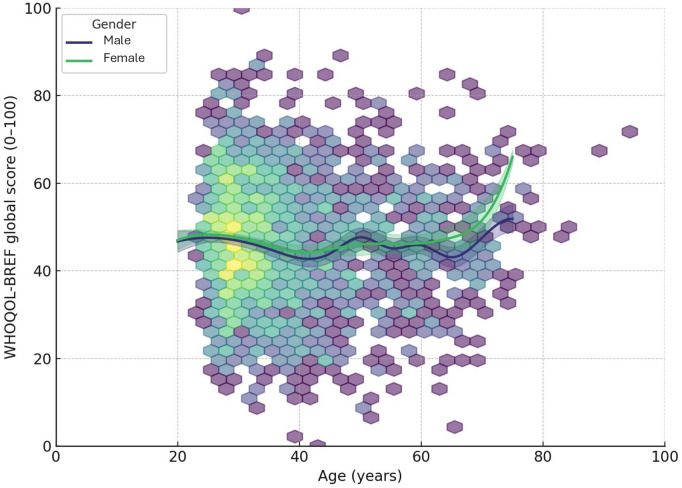
U-shaped age-score association by gender. Estimated mean score by age, stratified by gender. Both men and women show a U-shaped trajectory—lower in early/mid-adulthood with improvement after midlife; women remain slightly lower for most ages, with convergence and steeper gains after ~70. Higher values indicate better status.

Female physicians reported higher stress-related impact on professional performance (65.9% vs. 52.6%) and were more likely to report stress-related medical errors (34.1% vs. 29.7%). Stress was more frequently perceived as detrimental to personal relationships among women (77.2% vs. 64.0%) and as having increased over the past year (49.0% vs. 44.1%). All observed differences were statistically significant, consistently indicating greater vulnerability among female physicians (Fig 2).

High work demands and dissatisfaction with the health system were identified as the main drivers of stress. Burnout was most strongly associated with excessive workload (67.2%), while anxiety (55.3%) and depression (53.5%) were also linked to these demands. Dissatisfaction with the health system was reported as a major factor across conditions (52.2% for burnout, 48.1% for anxiety, and 47.9% for depression). Other contributing factors included insufficient public investment, excessive bureaucracy, precarious working conditions, workplace violence, lack of recognition, and inadequate compensation.

### Lifestyle and well-being

Healthier lifestyle practices were variably adopted (Table 4): 55.7% of physicians reported consuming more fresh than processed foods, 41.7% engaged in regular physical activity, 36.8% considered themselves to be meeting professional development expectations, 11.4% participated in volunteer activities, and only 6.0% practiced meditation or mindfulness regularly. Lower levels of physical activity, poorer diet quality, unmet professional expectations, and lack of social engagement were consistently associated with higher prevalence of adverse mental health outcomes.

**Table 4:** Self-reported lifestyle and professional activities among physicians (n = 2005).

Activity	Percentage (%)
Consumes more fresh foods than processed products	55.7
Engages in regular physical activity (≥150 min/week)	41.7
Meets own expectations regarding professional development	36.8
Participates in volunteer activities (NGOs, religious, social, private, or independent)	11.4
Regularly practices meditation or mindfulness	6.0

### Multivariable predictor contributions

Weighted least squares regression models were fitted for each domain and the QoL score to assess unique variance contributions (Table 5). Mental disorder emerged as the dominant predictor of variance in the Psychological domain and the QoL score, accounting for the largest ΔR^2^ values and highly significant omnibus tests (*p* < 0.001). In the Social relations domain, sex was the strongest predictor, uniquely explaining a notable portion of the variance, while mental disorder also contributed. The Physical and Environment domains showed modest but significant contributions of age group and region, in addition to mental disorder. Career stage contributed negligibly across all models.

**Table 5:** Weighted WHOQOL-BREF scores (0–100) among Brazilian physicians, by sex, region, age group, career stage, and presence of mental disorder.

Domain	Sex (F–M diff)	Region (lowest–highest)	Age (youngest–oldest)	Career (shortest–longest)	Mental disorder (Yes–No diff)
Physical	–0.7 (ns)	North 52.9 – Southwest 56.5 (*p* < 0.05)	Younger ≤35 lower vs ≥ 56 (+3–4 pts, *p* < 0.05)	≤5 yrs lower vs > 20 yrs (+3–4 pts, *p* < 0.05)	–5 pts, *p* < 0.001, d = –0.40
Psychological	–1.6 (ns)	42.4–47.1 (ns)	No significant differences	No significant differences	–10 pts, *p* < 0.001, d = –0.70
Social relations	+6.0, *p* < 0.001, d = 0.30	47.9–52.8 (ns)	No significant differences	No significant differences	–4 pts, *p* < 0.001, d = –0.30
Environment	–1.5, *p* = 0.034, d = –0.10	North 59.2 – South 65.5 (*p* < 0.001)	Younger (26–35) lower vs ≥ 56 (+4–6 pts, *p* < 0.05)	No significant differences	–5 pts, *p* < 0.001, d = –0.35
QoL	+0.8 (ns)	46.1–47.6 (ns)	No significant differences	No significant differences	–9 pts, *p* < 0.001, d = –0.65

Weighted means and differences estimated using post-stratification (raking) to match national distributions of sex and region. Values represent weighted mean scores (0–100 scale). Reported differences are expressed as points estimates between groups with significance values (*p*) and Cohen’s *d* when applicable. Abbreviations: F–M diff, female minus male difference; ns, not significant; pts, points.

**Fig 3:**
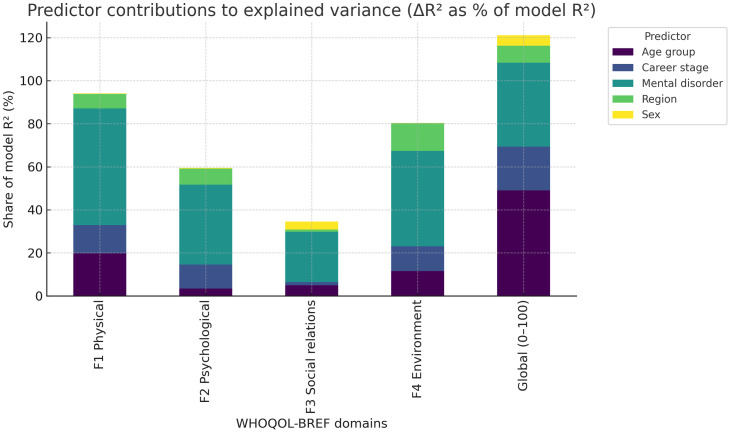
Predictor contributions to explained variance in WHOQOL-BREF domains and global QoL score. Relative contributions to explained variance for mental disorder, sex, age group, region, and career stage across WHOQOL-BREF domains and the global QoL score . Mental disorder explains the largest share for the Psychological domain and QoL; sex contributes most to Social relations; age group and region contribute modestly to Physical and Environment; career stage is minimal across outcomes. Estimates use post-stratified weights (raking by sex and region) to align the sample with national physician distributions.

A stacked bar chart (Fig 3) illustrates the proportion of model R^2^ uniquely attributable to each predictor. Mental disorder dominates in the Psychological and QoL outcomes, sex explains the largest share of variance in Social relations, and region and age group contribute modestly to Physical and Environment domains.

## Discussion

The findings of this study provide a comprehensive overview of the quality of life (QoL) and mental health of physicians in Brazil, offering unprecedented national estimates adjusted for sex and region. These results must be interpreted in the context of significant demographic and structural shifts in Brazilian medicine. In Brazil, the profession has undergone rapid “feminisation”: women constituted 46.6% of all physicians in 2022, and they were a majority (61.9%) among those aged ≤29 years, compared with only 25.2% in the group aged ≥60 years [4]. Despite this demographic advance, gender inequalities persist in specialty distribution and remuneration, with women predominating in Pediatrics (72.8%) and Obstetrics and Gynecology (67.6%) and men in General Surgery (80.1%) and Orthopedics (89.3%). Even after adjusting for specialty and age, women earned only 76% of the average male income in 2020, reflecting structural barriers in the medical labor market [21]. Geographic inequalities exacerbate these challenges, with greater physician density in metropolitan Southeast and South and shortages in the North and rural regions [4]

The weighted WHOQOL-BREF profile for Brazilian physicians — Physical 62.19, Psychological 55.46, Social Relations 56.52, Environment 63.64, global QoL score 59.45 — was consistently lower than population norms derived from general Brazilian samples, which typically report higher scores in physical and social domains [3]. When compared to other physician cohorts, these values were also below those of regional studies, such as Minas Gerais (Physical 69.65, Psychological 64.11, Social 60.81, Environment 66.77) [18]. International comparisons reinforce this interpretation: Egyptian physicians presented lower Environment scores (~44 after 0–100 conversion), showing that this domain is particularly sensitive to structural and systemic conditions [22]. Iranian healthcare workers also reported higher physical but lower environment scores, with similar vulnerability of psychological well-being [15].

A U-shaped trajectory was observed in overall QoL and domain scores across age groups, with lower scores among young and mid-career physicians and recovery in older age. This pattern reflects cumulative work stress, lack of autonomy, and lower institutional support early in career, with gradual improvements as professional control increases. Comparable life-course patterns were documented in international studies, with older physicians reporting better physical and environmental well-being but also possible survival bias, as healthier individuals remain active longer [23,24]. During the COVID-19 pandemic, however, declines in all domains were recorded among frontline workers, particularly younger physicians, due to long hours, secondary traumatization, and lack of institutional protection [19,25,26]

The Table 6 compiles international and national studies with WHOQOL-BREF scores transformed to the 0–100 scale. In Brazil, the present study with 1,999 physicians (weighted sample) revealed relatively low scores, especially in the psychological (55.5) and social (56.5) domains, with a weighted global QoL score of 59.5. In Germany, Sand et al.[27] analyzed 478 pre-hospital emergency physicians and found high scores across all domains, particularly in the environment (78.1) and physical (78.6) domains, findings consistent with more favorable structural conditions. In Saudi Arabia, AlAhmari et al.[28], studying 260 medical residents, reported a pattern closer to that observed in Brazil, with physical (59.8), psychological (54.5), and social (55.5) scores at comparable levels, highlighting that residents in high-income countries also experience psychological vulnerability. In Egypt, researchers [22] found low scores across all domains, especially in the environment (43.8), reflecting systemic precariousness. In contrast, West et al. [29] reported normative population scores for Australia between 72 and 76 in all domains, consistently higher than those observed among Brazilian physicians.

**Table 6:** WHOQOL-BREF domain scores among physicians and general populations across different income levels.

Table 6.A.							
Year	Country	Income level (WB)	Population	Physical	Psychological	Social	Environment
2024	Brazil	Upper-middle	Physicians (licensed), national sample	62.19	55.46	56.52	63.64
2016	Germany	High-income	Emergency physicians (pre-hospital)	78.6	72.1	67.5	78.1
2023	Saudi Arabia	High-income	Medical residents	59.8	54.5	55.5	62.5
2021	Egypt	Lower-middle	Physicians (various specialties)	55.8	51.8	55.4	43.8
2023	United States	High-income	General adult population	74.52	72.07	72.87	76.41
2006	Australia	High-income	General adult population	73.5	70.6	71.5	75.1
Table 6.B.							
**Grouped Analysis by Country Income Level**				**Physical**	**Psychological**	**Social**	**Environment**
**High-income**				71.6	67.3	66.8	73.0
**Lower-middle**				55.8	51.8	55.4	43.8
**Upper-middle**				62.2	55.5	56.5	63.6

Although not directly comparable, normative values from high-income countries such as Australia reinforce this contrast. Typical population scores in these countries exceed 70 across all domains, showing that Brazilian physicians report worse quality of life than the general population of high-income countries.

When grouping studies by World Bank income classification, a socioeconomic gradient becomes clear. In high-income countries such as Germany, Saudi Arabia, the United States, and Australia, mean scores are around 71.6 in the physical domain, 67.3 in the psychological domain, 66.8 in the social domain, and 73.0 in the environment domain. Brazil, classified as an upper-middle-income country, presented lower values, with 62.2, 55.5, 56.5, and 63.6, respectively. Egypt, a lower-middle-income country, presented the lowest results, with 55.8, 51.8, 55.4, and 43.8 in the same domains. This gradient shows a clear association between national income level and higher mean quality-of-life scores, particularly in the physical and environment domains.

The comparative analysis confirms that Brazilian physicians report lower quality of life not only relative to the general population of high-income countries but also when compared with peers in developed medical contexts, such as Germany. The Brazilian pattern more closely resembles that observed among residents in Gulf countries, such as Saudi Arabia, suggesting that career stage and working conditions play a role as important as national income level. Egypt emerges as the lower extreme, with marked impairment in the environment domain, which underscores the influence of systemic factors on quality-of-life perceptions. This finding is consistent with the literature showing that the environment domain of the WHOQOL-BREF is particularly sensitive to structural inequalities [15,28].

Grouping the results by World Bank income level reveals a consistent socioeconomic gradient, with high-income countries sustaining higher mean scores across all domains, while middle- and low-income countries display clear deficits. This pattern emphasizes that, beyond individual determinants such as sex, lifestyle, and career stage, macrostructural determinants strongly influence physicians’ well-being.

These findings support the interpretation that the quality of life of Brazilian physicians is shaped not only by internal inequalities of gender and region but also by broader global disparities in development. Such vulnerabilities may originate during medical training. Resende et al.[30] demonstrated a significant decline in quality of life and mental health among Brazilian medical students across the course of study, with the psychological and physical domains being most affected. These results underscore that the academic demands of medical education can profoundly compromise students’ well-being, highlighting the importance of implementing preventive strategies from the early stages of training.

By positioning Brazil between residents of high-income countries and professionals from lower-income settings, the study expands the understanding of this phenomenon and indicates that effective interventions must simultaneously address systemic factors, such as financing and working conditions; meso-level factors, such as institutional environments; and micro-level factors, such as lifestyle and social support.

Sex differences in WHOQOL-BREF were modest at the domain level: men scored slightly higher in Physical and Psychological domains, while women reported higher Social relations scores. However, the prevalence of diagnosed mental illness was significantly higher among women (46.8% vs. 33.5% in men). Depression (22.1%), anxiety (33.5%), and burnout (6.7%) were common, mirroring findings from international literature where female physicians consistently report higher emotional exhaustion, more harassment, and greater work–family conflict [6,7,31,32]. These apparent contradictions between QoL domains and psychiatric morbidity may be explained by the *dynamic reference point* theory, which suggests that individuals recalibrate expectations in response to structural barriers. Women may emphasize social and relational fulfillment as compensatory sources of QoL, even in the presence of higher psychiatric burden [32]

High and stable scores in Social relations across age groups highlight the protective effect of social networks in medicine. Older women scored particularly high in this domain, consistent with studies emphasizing collegiality and peer support as buffers against burnout [18]. Environmental scores, although higher than in low-resource countries, were strongly associated with income quartiles and regional differences, underscoring the contextual nature of this domain.

Lifestyle factors emerged as important correlates of mental health. Only 41.7% of physicians reported regular physical activity and 55.7% reported consumption of mostly fresh foods. Evidence from prospective studies indicates that physicians who engage in healthy lifestyle practices report lower stress and better mental health outcomes [33]. Interventions promoting exercise, balanced diet, and mindfulness may therefore be protective levers for professional well-being.

Unlike symptom-based screening tools such as the Depression Anxiety Stress Scale (DASS-21), which capture psychiatric morbidity, the WHOQOL-BREF assesses *subjective quality of life* in four interrelated domains (physical, psychological, social, environment). This broader construct allows identification of systemic and occupational determinants beyond psychopathology [13]. Without such measures, diagnostic prevalence relies on self-report, risking underestimation or recall bias. Nonetheless, the WHOQOL-BREF’s transcultural validation in 23 countries and its consistent psychometric reliability support its use in large, heterogeneous physician samples, allowing international comparability [13].

The methodological strengths of this study include its large national sample, post-stratification weighting for sex and region, use of a validated transcultural instrument (WHOQOL-BREF), and multivariable models assessing unique contributions of sex, region, age, career stage, and mental disorders. Together, these features enhance external validity and situate findings within both national and international comparative frameworks.

### Strengths and limitations

Strengths of this study include its large, nationally weighted sample; use of a validated transcultural QoL instrument (WHOQOL-BREF); rigorous post-stratification procedures; and multivariable models assessing unique contributions of demographic and clinical factors. These features enhance external validity and allow comparisons with both national and international cohorts.

However, several limitations must be acknowledged. First, the cross-sectional design precludes causal inference. Second, the reliance on self-reported diagnoses may introduce recall and social desirability bias, with potential under- or over-estimation of mental health conditions, perception of QoL and healthy lifestyle behavior. Third, although post-stratification mitigated some sampling imbalances, physicians in rural and underserved regions remain underrepresented. Fourth, potential confounders such as comorbities, public vs. private sector practice, and workload distribution by specialty were not fully captured. Finally, regression models yielded low R^2^ values, reflecting the multifactorial nature of physician QoL and the limited explanatory power of demographic variables alone. In addition, the use of WHOQOL-brief reported as single a score as the mean of the four domain scores may limit direct comparisons because this is not the standard reporting approach for the instrument. Thus, direct comparisons with studies using domain-level reporting or other scoring methods should be made cautiously

### Implications and Future Directions

Our findings have several implications. Interventions to improve physician well-being must address both structural inequalities (gender pay gap, regional disparities, precarious work conditions) and individual-level factors (promotion of healthy lifestyles, stress management, and peer support networks). Policy makers should prioritize equitable distribution of physicians, investment in safer work environments, and gender-sensitive strategies in medical workforce planning.

Future research should adopt longitudinal designs to clarify causal pathways between occupational stressors, mental disorders, and QoL trajectories. Incorporating standardized psychiatric screening tools (e.g., DASS-21, PHQ-9, GAD-7) alongside QoL measures may improve comparability and diagnostic accuracy. In addition, developing psychometric methods to aggregate QoL into a single multidimensional index may improve interpretability, simplify analyses in future studies, strengthen comparability across populations, and inform the development and evaluation of public policies. Finally, intervention trials testing institutional and individual strategies (e.g., reduced workloads, mentorship programs, mindfulness-based interventions) are needed to guide actionable reforms.

## Conclusion

Brazilian physicians report lower QoL than the general population, with marked vulnerabilities in psychological well-being and strong associations with mental disorders. Gender disparities persist, with women experiencing higher psychiatric morbidity despite preserved social relations. Structural inequalities, workload, and dissatisfaction with the health system are key drivers of stress and reduced QoL. Addressing these challenges requires integrated strategies that combine structural reforms, gender equity policies, and lifestyle-promoting interventions.

## References

[1] The WHOQOL Group. Development of the World Health Organization WHOQOL-BREF quality of life assessment. Psychol Med. 1998;283(3):551–8. doi: 10.1017/S0033291798006667 9626712

[2] Fleck MP, Louzada S, Xavier M, Chachamovich E, Vieira G, Santos L, et al. Application of the Portuguese version of the abbreviated instrument of quality life WHOQOL-bref. Rev Saude Publica. 2000;34(2):178–83. doi: 10.1590/s0034-89102000000200012 10881154

[3] Cruz LN, Polanczyk CA, Camey SA, Hoffmann JF, Fleck MP. Quality of life in Brazil: normative values for the WHOQOL-bref in a southern general population sample. Qual Life Res. 2011;20(7):1123–9. doi: 10.1007/s11136-011-9845-3 21279448

[4] Scheffer M, Almeida CJ, Cassenote AJF, Dias IWH, Moreira JPL, Sousa J, et al. Demografia Médica no Brasil 2025. Brasília: Ministério da Saúde; São Paulo: Faculdade de Medicina da Universidade de São Paulo; 2025.

[5] Mainardi GM, Cassenote AJF, Guilloux AGA, Miotto BA, Scheffer MC. What explains wage differences between male and female Brazilian physicians? A cross-sectional nationwide study. BMJ Open. 2019;9(4):e023811. doi: 10.1136/bmjopen-2018-023811 31048423 PMC6502025

[6] Obeng Nkrumah S, Adu MK, Agyapong B, da Luz Dias R, Agyapong VIO. Prevalence and correlates of depression, anxiety, and burnout among physicians and postgraduate medical trainees: a scoping review of recent literature. Front Public Health. 2025;13:1537108. doi: 10.3389/fpubh.2025.1537108 40697832 PMC12279716

[7] Jefferson L, Bloor K, Maynard A. Women in medicine: historical perspectives and recent trends. Br Med Bull. 2015;114(1):5–15. doi: 10.1093/bmb/ldv007 25755293

[8] dos Santos HA, Segundo JM, Barreto MLL, dos Santos VR, de Azevedo GD, de Sousa ACP. Factors associated with medical students’ quality of life in a Brazilian northeast countryside university. Rev Bras Educ Med. 2021;45(3):e167. doi: 10.1590/1981-5271v45.3-20210042.ing

[9] Auttama N, Seangpraw K, Ong-Artborirak P, Tonchoy P. Factors Associated with Self-Esteem, Resilience, Mental Health, and Psychological Self-Care Among University Students in Northern Thailand. J Multidiscip Healthc. 2021;14:1213–21. doi: 10.2147/JMDH.S308076 34079280 PMC8166326

[10] Bernhard J, Lowy A, Maibach R, Hürny C. Response shift in the perception of health for utility evaluation. European Journal of Cancer. 2001;37(14):1729–35. doi: 10.1016/s0959-8049(01)00196-4 11549425

[11] Vutyavanich T, Sreshthaputra R, Thitadilok W, Sukcharoen N. Quality of life and risk factors that affect the quality of life of Thai female physicians. J Med Assoc Thai. 2007;90(11):2260–5. 18181304

[12] Tran ANP, To QG, Huynh V-AN, Le KM, To KG. Professional quality of life and its associated factors among Vietnamese doctors and nurses. BMC Health Serv Res. 2023;23(1):924. doi: 10.1186/s12913-023-09908-4 37649084 PMC10469419

[13] Skevington SM, Lotfy M, O’Connell KA, WHOQOL Group. The World Health Organization’s WHOQOL-BREF quality of life assessment: psychometric properties and results of the international field trial. A report from the WHOQOL group. Qual Life Res. 2004;13(2):299–310. doi: 10.1023/B:QURE.0000018486.91360.00 15085902

[14] Cheung YB, Yeo KK, Chong KJ, Khoo EY, Wee HL. Reliability and Validity of the English-, Chinese- and Malay-Language Versions of the World Health Organization Quality of Life (WHOQOL-BREF) Questionnaire in Singapore. Ann Acad Med Singap. 2017;46(12):461–9. doi: 10.47102/annals-acadmedsg.v46n12p461 29355283

[15] Gholami A, Jahromi LM, Zarei E, Dehghan A. Application of WHOQOL-BREF in Measuring Quality of Life in Health-Care Staff. Int J Prev Med. 2013;4(7):809–17. 24049600 PMC3775221

[16] Castro MML, Hökerberg YHM, Passos SRL. Validade dimensional do instrumento de qualidade de vida WHOQOL-BREF aplicado a trabalhadores de saúde. Cad Saude Publica. 2013;29(7):1357–69. doi: 10.1590/s0102-311x2013000700010 23843003

[17] Kluthcovsky ACGC, Kluthcovsky FA. O WHOQOL-bref, um instrumento para avaliar qualidade de vida: uma revisão sistemática. Rev psiquiatr Rio Gd Sul. 2009;31(3 suppl). doi: 10.1590/s0101-81082009000400007

[18] Moreira WC de A, de-Souza FT, Dias EC, Gomes SA, da-Silva MG, Gomes ACQ, et al. Quality of life of physicians in the state of Minas Gerais, Brazil. Rev Bras Med Trab. 2022;20(3):375–86. doi: 10.47626/1679-4435-2021-730 36793470 PMC9904827

[19] Serrão C, Martins V, Ribeiro C, Maia P, Pinho R, Teixeira A, et al. Professional Quality of Life Among Physicians and Nurses Working in Portuguese Hospitals During the Third Wave of the COVID-19 Pandemic. Front Psychol. 2022;13:814109. doi: 10.3389/fpsyg.2022.814109 35178016 PMC8845595

[20] Gobbo M Jr. Dataset of Impacts of gender disparities in mental health and quality of life: a cross-sectional study of Brazilian physicians. Zenodo. 2025. doi: 10.5281/zenodo.17898332 PMC12795389 41525341

[21] Scheffer M, Ribeiro FOP, Poz MD, Andrietta L. Physicians’ income in Brazil: a study on information sources. Rev Assoc Med Bras (1992). 2022;68(5):691–6. doi: 10.1590/1806-9282.20220172 35584498

[22] Abdelrahman AI, Goda NA, Bendary MM. Quality of life among physicians in Egypt and its influencing factors: a cross-sectional study. Int J Public Health Sci. 2024;13(4):1696–703. doi: 10.11591/ijphs.v13i4.23946

[23] Dyrbye LN, Satele D, Sloan J, Shanafelt TD. Ability of the physician well-being index to identify residents in distress. J Grad Med Educ. 2014;6(1):78–84. doi: 10.4300/JGME-D-13-00117.1 24701315 PMC3963800

[24] Lall MD, Gaeta TJ, Chung AS, Chinai SA, Garg M, Husain A, et al. Assessment of Physician Well-being, Part Two: Beyond Burnout. West J Emerg Med. 2019;20(2):291–304. doi: 10.5811/westjem.2019.1.39666 30881549 PMC6404719

[25] Archuleta S, Ibrahim H, Pereira TL-B, Shorey S. Microaggression Interactions Among Healthcare Professionals, Trainees and Students in the Clinical Environment: A Mixed-Studies Review. Trauma Violence Abuse. 2024;25(5):3843–71. doi: 10.1177/15248380241265380 39082181

[26] Oliveira MML da S, Butrico GF de O, Vila V da SC, Moraes KL, Rezende MAD, Santos LTZ, et al. Quality of life at work for health professionals during the COVID-19 pandemic. Rev Bras Enferm. 2024;77(Suppl 1):e20230461. doi: 10.1590/0034-7167-2023-0461 38958357 PMC11213535

[27] Sand M, Hessam S, Bechara FG, Sand D, Vorstius C, Bromba M, et al. A pilot study of quality of life in German prehospital emergency care physicians. J Res Med Sci. 2016;21:133. doi: 10.4103/1735-1995.196615 28331519 PMC5348825

[28] AlAhmari FS, Aloqail A, Almansour S, Bagha M. State of well-being among residents in a tertiary center in Riyadh, Saudi Arabia. BMC Med Educ. 2023;23(1):655. doi: 10.1186/s12909-023-04596-4 37684578 PMC10492334

[29] West EC, Williams LJ, Stuart AL, Pasco JA. Quality of life in south-eastern Australia: normative values for the WHOQOL-BREF in a population-based sample of adults. BMJ Open. 2023;13(12):e073556. doi: 10.1136/bmjopen-2023-073556 38072488 PMC10729265

[30] Silveira de Resende M, Santos IM, de Moura EC, de Almeida Pedro R, Gobbo M Jr. Impact of medical school on quality of life and mental health in Brazil: a cross-sectional comparative study. BMJ Open. 2025;15(6):e097917. doi: 10.1136/bmjopen-2024-097917 PMC12142097 40467311

[31] Mathews E, Hammarlund R, Kullar R, Mulligan L, Le T, Lauve S, et al. Sexual Harassment in the House of Medicine and Correlations to Burnout: A Cross-Sectional Survey. Ochsner J. 2019;19(4):329–39. doi: 10.31486/toj.19.0019 31903056 PMC6928669

[32] Amiri S, Mahmood N, Mustafa H, Javaid SF, Khan MA. Occupational Risk Factors for Burnout Syndrome Among Healthcare Professionals: A Global Systematic Review and Meta-Analysis. Int J Environ Res Public Health. 2024;21(12):1583. doi: 10.3390/ijerph21121583 39767426 PMC11675210

[33] Frank E, Zhao Z, Fang Y, Rotenstein LS, Sen S, Guille C. Experiences of Work-Family Conflict and Mental Health Symptoms by Gender Among Physician Parents During the COVID-19 Pandemic. JAMA Netw Open. 2021;4(11):e2134315. doi: 10.1001/jamanetworkopen.2021.34315 34767022 PMC8590168

